# Genetic Testing on Patients with Developmental Delay: A Preliminary Study from the Perspective of Physicians

**DOI:** 10.3390/healthcare10071236

**Published:** 2022-07-02

**Authors:** Gwanwook Bang, Sook Joung Lee, Bomyee Lee, Minji Park, So-Youn Park

**Affiliations:** 1 Department of Medical Education and Humanities, School of Medicine, Kyung Hee University, Seoul 02447, Korea; alchemy_tc@khu.ac.kr (G.B.); parkmj9315@gmail.com (M.P.); 2 Department of Physical Medicine & Rehabilitation, Daejeon St. Mary’s Hospital, College of Medicine, The Catholic University of Korea, Seoul 06591, Korea; lsj995414@hanmail.net; 3 Korea National Institute for Bioethics Policy, Seoul 04522, Korea; bylee@nibp.kr

**Keywords:** disability, genetic testing, informed consent, medical communication, clinical decision-making

## Abstract

This study aimed to discover and propose solutions to various decision-making problems, including obtaining consent, encountered by physicians when administering genetic testing to patients with disabilities. A preliminary survey and focus group interviews (FGIs) were conducted with 27 specialists who had 5–25 years of clinical experience in rehabilitation medicine, pediatrics, and obstetrics and gynecology, regarding their experience in providing genetic testing to people with developmental disabilities. This included the “role of medical staff in the patient’s decision-making process”, “difficulty of the consent process for genetic testing”, and so forth. Some limitations were identified in the genetic testing communication process for patients with disabilities. Although providing information corresponding to the level of understanding of each person and accurately evaluating the correct consenting ability is important, the usage rate of auxiliary tools, such as booklets and videos, was only 50.0%. Additionally, there were concerns regarding the marriage prospects of people with disabilities. For people with developmental disabilities to provide consent for genetic testing, legal consent forms and explanation aids that consider individual characteristics are necessary. Moreover, education on disability awareness throughout society, including cost support, is needed.

## 1. Introduction

Modern society has entered the fourth industrial revolution, presenting new opportunities in the medical field. Rapid advancements in medical technology have led to the introduction of innovative methods of diagnosing and treating diseases or disorders with previously unidentifiable causes [1].

Since the enactment and enforcement of the new medical technology evaluation law in 2007 [2], numerous studies have been conducted in Korea to generalize precision medicine or customize diagnosis and treatment according to individual characteristics. Specifically, innovations in genetic testing, such as next-generation sequencing (NGS) and direct-to-consumer (DTC) models, have made precision medicine possible, providing people with the opportunity to lead a healthy life. Moreover, people with developmental disabilities, who have suffered due to genetic problems, are the most likely to be affected by these changes [3].

Disability is defined as “difficulty encountered as a result of impairment, activity limitation, or participation restriction” [4]. Disability is not only caused by personal impairment but also by problems such as restricted service use and lack of participation in social systems. These problems stem from the interaction between environmental factors, such as policies, and personal factors, including self-esteem.

People with disabilities face various barriers, including social stigma and restricted mobility. Moreover, individuals with developmental disabilities, who face various constraints regarding communication and access to medical services, require greater attention and assistance to live a healthy life [5]. Genetic testing has advanced rapidly in recent years and could help resolve the problems experienced by people with disabilities. Genetic testing has garnered great interest, enabling the diagnosis and treatment of previously unidentified disorders by determining their cause via DNA analysis and other methods. If genetic testing is utilized properly, it will be the most important tool that supports the planning of health promotion initiatives for people with developmental disabilities [6].

However, despite these positive effects, if people with developmental disabilities cannot easily access or use this technology, then the gap between people with and without disabilities will widen. Therefore, to guarantee the benefits of medical technology for people with developmental disabilities, social and institutional devices should be tailored to the characteristics of people with developmental disabilities. This will allow them to access and properly utilize the relevant medical services. If these existing problems are resolved, people with developmental disabilities will be able to exercise voluntary decision-making and maximize participation in the process of receiving new medical technology [6,7,8].

In December 2017, the “Act on Guarantee of Right to Health and Access to Medical Services for Persons with Disabilities” (hereinafter, Act of H&APWD) was enacted in Korea. The Act of H&APWD stipulates that access to healthcare services for persons with disabilities should be improved to ensure equality, regardless of the existence of a disability, type and degree of the disability, and gender. This resulted in the establishment of a national system to ensure that persons with disabilities have not only the right but also equal access to optimal healthcare and protection. This act is currently being recommended at the national and local levels by the Korean government [9].

Moreover, 152 countries participated in the UN’s “Convention on the Rights of Persons with Disabilities”. The convention emphasized the importance of new medical technology to increase the independence and social participation of people with disabilities, calling for national investments and efforts to ensure accessibility. Furthermore, information on new technologies will increase the accessibility of systems and services to people with disabilities. This will help maintain their freedom to accept information, express opinions, and receive rehabilitation. Therefore, to realize the healthcare rights of people with disabilities, accurate and prompt access to information on new medical technologies is necessary [10].

Genetic testing is sought after by people with developmental disabilities. However, they may lack knowledge about genetic testing and have limited access to relevant information. The process of obtaining consent is necessary for cooperative decision-making between patients and doctors in patient-centered medical care. However, few studies have investigated the difficulties faced by people with developmental disabilities while undergoing genetic testing. Furthermore, few studies have determined whether the process for rational decision-making regarding genetic testing is applicable to people with developmental disabilities [11].

Therefore, we conducted a preliminary study on the experiences of medical staff conducting genetic testing on people with developmental disabilities. We aimed to suggest measures to resolve the issues related to performing genetic testing at the actual treatment site.

## 2. Method

### 2.1. Participants

This study was conducted among 27 medical doctors who had experience in recommending or performing genetic testing while providing healthcare for patients with developmental delays. The number of participants was set considering the minimum number required for a typical preliminary study, which ranges between 12 and 30 participants [12].

### 2.2. Study Tools

In order to investigate the obstacles faced by doctors and their process of performing genetic tests on patients with disabilities, a questionnaire was developed based on a review of previous studies [13,14,15,16,17]. It comprised 30 items, including eight open-ended items on “general characteristics” and three items on medical specialization, clinical experience, and the “experience of providing medical service on genetic testing”. There were 19 multiple-choice items rated on a 5-point Likert scale that assessed the “degree of helpfulness in genetic testing for persons with disabilities” (3 items), “level of genetic testing process” (7 items), and “level of concern when conducting genetic testing” (9 items). The content validity of the questionnaire was verified following a review by three medical experts. An internal consistency test was performed on the 19 multiple-choice items. The results revealed that Cronbach’s α for “the degree of help of genetic testing for the disabled”, “the degree of the process of performing genetic testing”, and “the level of concern when conducting genetic testing” was 0.707, 0.933, and 0.737, respectively. IBM SPSS version 26.0 (IBM Corp., Armonk, NY, USA) was used for statistical analysis.

### 2.3. Analysis Method

The survey was analyzed by dividing the items into multiple-choice and open-ended questions. The multiple-choice questions were examined through frequency analysis, while the open-ended type questions were examined using a qualitative analysis method. Three expert researchers conducted the qualitative analysis after sufficient discussion. 

### 2.4. Ethical Consideration

In order to protect the privacy of the study participants, no personally identifiable information (e.g., name and affiliation) was collected. Furthermore, the questionnaire was only administered to those who provided voluntary consent for study participation after receiving an explanation of the study’s purposes. This study was conducted after receiving approval from the Institutional Review Board of Kyung Hee University (approval no. KHSIRB-22-010(RA)).

## 3. Results

### 3.1. General Characteristics

All 27 study participants diligently responded to the questionnaire. The participants majored in rehabilitation medicine (16; 59.3%), pediatrics (9; 33.3%), and gynecology (2; 7.4%). Participants’ general characteristics, including their selected majors, specializations, and years of experience, are presented in Table 1.

### 3.2. Clinical Experience with Genetic Testing

Participants were asked if they had any experience providing genetic testing and responded “yes/no” to four questionnaire items. They could also provide a brief explanation of their responses to some questions. Most of their responses were positive (Table 2).

### 3.3. Usefulness of Genetic Testing When Treating the Developmentally Disabled

Participants rated the usefulness of genetic testing for patients with developmental delay on a 5-point Likert scale (1 = not helpful at all, 5 = very helpful). All items received positive responses (Table 3).

### 3.4. Concerns Regarding the Provision of Genetic Testing Services

Factors that should be considered while conducting genetic testing of patients with developmental delay were discussed in previous studies [13,14,15,16,17]; the researchers developed the questionnaire items based on these findings [13,14,15,16,17]. The respondents’ level of concern for each factor was rated on a 5-point Likert scale (1 = not concerned at all; 5 = very concerned). The items were open-ended and received short or descriptive answers.

**Figure 1:**
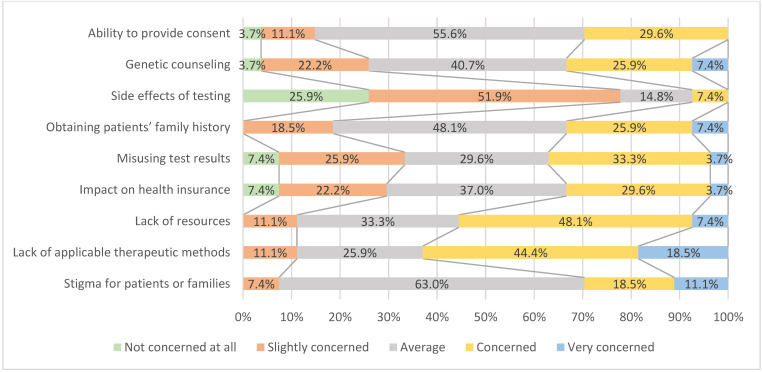
Concerns about genetic testing services.

In addition, physicians were asked how concerned they were about the following items in the actual clinical setting. The developed items as the results are presented in Figure 1.

Most respondents (77.8%) were “not concerned at all” or “slightly concerned” about the “side effects of testing”. In total, respondents who were “concerned” or “very concerned” about the “lack of resources” and “lack of applicable therapeutic method” were 55.5% and 62.9%, respectively. Meanwhile, 37.0%, 33.3%, and 33.3% of participants were “concerned” or “very concerned” about “misusing test results”, “impact on health insurance”, and “genetic counseling”, respectively. Some respondents were not concerned about issues regarding the “ability to provide consent”, “genetic counseling”, “side effects of testing”, “misusing test results”, and “impact on health insurance”.

### 3.5. Implementation of Each Step When Conducting Genetic Testing 

The implementation items were developed based on previous studies [13,14,15,16,17] by considering the steps the physician needed to perform while conducting genetic testing on patients with developmental delay. The 25 physicians who recommended genetic testing to patients with developmental delay in the past year responded to the items evaluating the degree to which each step was implemented when conducting genetic testing. The items were rated on a 5-point Likert scale (1 = not performed at all; 5 = completely performed). 

**Figure 2:**
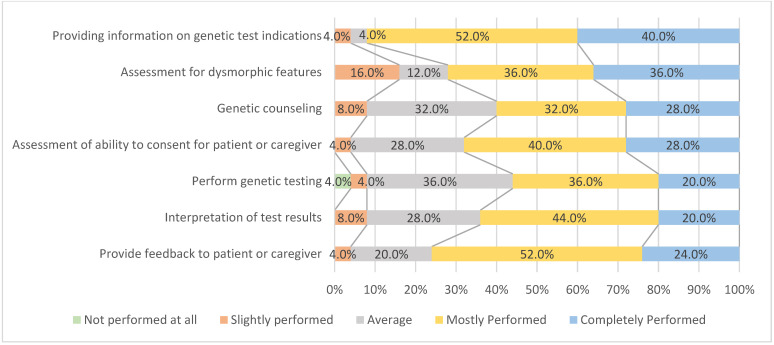
Implementation of each process when providing genetic testing service.

The following items were thus formulated: “providing information on indications for genetic testing”, “assessment for dysmorphic features”, “genetic counseling”, “assessment of the patients’ or caregivers’ ability to provide consent”, “conducting genetic testing”, “interpretation of test results”, and “providing feedback to patients or caregivers”. The degree of physicians’ implementation of each item is depicted in Figure 2. 

Overall, each step was diligently performed while conducting genetic testing. However, the percentage differed by item for those who responded: “performed (slightly, mostly)”. All items were performed well, considering the percentage of “mostly performed” and “completely performed” responses: “providing information on indications for genetic testing” (92.0%), “assessment for dysmorphic features” (72.0%), “genetic counseling” (60.0%), “assessment of the patients’ or caregivers’ ability to provide consent” (68.0%), “conducting genetic testing” (56.0%), “interpretation of test results” (64.0%), and “providing feedback to patients or caregivers” (76.0%). Meanwhile, regarding “perform genetic testing”, 4.0% of respondents answered, “not performed at all”. 

### 3.6. Reasons for Recommending Genetic Testing and Obstacles Faced

We investigated the reasons physicians recommended genetic testing for patients with developmental delay and identified the obstacles they faced. Some of the responses given during the FGIs are described below.

#### 3.6.1. Reasons for Recommending Genetic Testing for People with Developmental Disabilities

(1) Predict, prepare, and family planning

*“If you know the name of the disease of the child, you can predict and prepare for the child’s future. There are certain genetic diseases that have a specific treatment method, so testing is absolutely necessary. It is also important for additional family planning.”*

*(Participant 11)*

*“First, if confirmed through genetic testing, comorbidities can be diagnosed and treated early. Second, the prognosis of the patient can be identified so that medical resource allocation can be discussed. Third, it can predict hereditary potential and serve as a resource for genetic counseling in case the patient or their relative has a child.”*

*(Participant 20)*

(2) Child rearing

*“When a child has symptoms such as developmental delay, motor abnormality, or muscle weakness of unknown cause, a diagnosis can be determined via genetic testing. Accordingly, medications that can control the symptoms can be prescribed. In addition, genetic testing can provide the basis for preparing for a future at home, once the prognosis is determined, in terms of how much can be achieved after understanding the circumstances for rehabilitation. All this can be determined merely in response to a child falling behind at school. So, I always recommend genetic testing.”*

*(Participant 24)*

#### 3.6.2. Obstacles Faced in Recommending Genetic Testing to Patients with Disabilities

(1) High cost and lack of auxiliary tools

*“The high cost of the test and the patient’s economic status may result in hesitation to undergo the test. I think the explanation of the test is conveyed mostly through the informed consent form, and an easy-to-understand information sheet is needed.”*

*(Participant 1)*

*“Chromosomal microarray analysis, which is performed as a primary test for developmental delay, may be covered by insurance, but there is the economic burden of additional tests that may need to be performed. The lack of understanding by the parents about genetic testing and the lack of auxiliary explanatory tools may be reasons for their hesitance.”*

*(Participant 13)*

*“Reasons for hesitance among disabled patients could include their economic status, the degree of understanding of the caregiver, lack of proper explanation, lack of auxiliary tools, lack of time, and lack of compensation for the time taken for proper explanation by the medical staff, and lack of experts who can provide repeated explanations.”*

*(Participant 16)*

(2) Stigmatizing effect

*“Parents are concerned that disability may affect their children’s prospects of marriage (stigma effect). First, elderly patients or their spouses are often afraid of confirmation through genetic testing due to concerns about their children’s marriage. Second, young adult patients also often postpone genetic testing until they are married. Third, in the case of patients with disabilities, if the test is not covered by insurance, the cost burden is high. Fourth, although there are various genetic diseases, it is difficult to obtain auxiliary tools, such as well-written brochures, with which patients can explain to their caregivers the purpose and benefits of genetic tests.”*

*(Participant 21)*

## 4. Discussion

This study aimed to discover and propose solutions for various on-site decision-making problems encountered by physicians in administering genetic testing to persons with disabilities. These problems include obtaining consent and voluntary participation in decision-making. A preliminary survey was thus conducted among physicians with experience in treating individuals with developmental disabilities.

Our findings revealed that various considerations were necessary when conducting genetic testing on people with developmental disabilities in the treatment setting. For various reasons, the physicians’ levels of concern differed regarding the problems encountered. There were also differences between some of the items under implementation. This may have emerged from issues related to the environment outside the treatment setting, as well as the diagnostic process, such as a lack of communication tools, lack of materials, and high medical costs.

Few studies have examined the access of persons with disabilities to healthcare services and the genetic testing experience among this population. Conversely, however, this population will benefit the most from genetic testing, which is now covered by insurance or DTC projects recently promoted by the government. 

Our findings indicated that the reasons for conducting genetic testing on people with developmental disabilities included determining the diagnosis and treatment with the aim of identifying the cause of congenital disability in the individual’s DNA. Another reason was for family planning at the request of patients with disabilities to identify the likelihood of potential disability in their future children. 

The purpose of genetic testing is to provide a better quality of life for patients with disabilities. However, when conducting genetic testing on this population, the medical staff must consider the various issues identified in this study (see Figure 1). However, in the future, before interpreting these issues, an in-depth discussion is necessary to determine the concerns of medical staff. For example, there may be differences in not only the side effects of different genetic tests but also the severity of developmental disabilities, which may impact patients’ ability to provide consent. 

Moreover, the difficulties experienced by physicians while conducting genetic testing on patients with disabilities can be largely divided into problems during and beyond the diagnostic process. Regarding problems during the diagnostic process, 27 physicians identified various obstacles to each step of the genetic testing process. In terms of clinical aspects, providing information on genetic testing, assessment for dysmorphic features, and providing test feedback had a high implementation rate. However, genetic counseling and the assessment of the patients’ or caregivers’ ability to provide consent showed a relatively lower rate of implementation among some physicians. Patients with developmental delay may lack understanding due to their impaired cognitive ability; thus, their ability to provide consent should be assessed to determine whether they can adequately understand and consent to the test. Simultaneously, information should be provided that is suited to their level of understanding. If persons with disabilities cannot provide consent, then their caregivers can usually provide consent on their behalf when making a decision that involves the selection of medical services [18]. However, unless the patient is unable to provide consent due to severe cognitive impairment, the patient must be able to exercise their right to decide to undergo genetic testing based on sufficient understanding, similarly to non-disabled patients [19]. Therefore, physicians must assess the patient’s ability to provide consent and determine whether patients with disabilities can sufficiently understand genetic testing and make an informed decision.

Problems beyond the diagnostic process include a lack of communication tools that can supplement sufficient explanation on genetic testing for the patients with developmental disabilities or a lack of materials that can aid their understanding. Thus, problems in the treatment setting mentioned above do not arise because of physicians’ inabilities to assess patients’ ability to provide consent. As demonstrated by our results, the currently used legal forms [20,21] in relation to genetic testing do not cater to the characteristics of people with developmental disabilities who have difficulty with communication. Moreover, communication tools to enhance explanation during the diagnostic process are lacking. The currently available informed consent form for genetic testing has a legally approved template; however, it is difficult for people with developmental disabilities to understand. Moreover, it is only available in paper format rather than audio or Braille formats and omits easy-to-understand explanations of genetic testing. Therefore, difficulties may arise in providing sufficient information to patients with sight and hearing disabilities. 

The counseling process and the difficulty physicians face in assessing patients’ ability to provide consent, as well as the lack of communication tools, present obstacles for patients with developmental disabilities and their families in understanding genetic testing. This ultimately hinders their ability to make decisions with sufficient information about their medical care.

Therefore, it is necessary to improve various systems for individuals with developmental disabilities while creating communication tools that consider their individual characteristics. This will enable them to exercise their right to make decisions in receiving genetic testing. First, communication tools and materials that are developed to cater to their level of understanding, the severity of their disability, and age must be made available. Furthermore, regarding genetic testing, a system that can communicate according to the characteristics of the disorder should be established. It will thus be necessary to understand disabilities and learn how to communicate with individuals with disabilities via the domestic medical curriculum (e.g., standardized patient education). Second, regarding problems beyond the diagnostic process, 55.5% of experts in this study responded that “lack of resources” was a major obstacle for patients with disabilities in using genetic testing. Compared to their able-bodied counterparts, people with disabilities have a relatively high burden of medical costs and are typically financially strained due to the loss of working ability. When the medical costs are not entirely covered by the government, the costs of genetic testing can be an additional burden. Although this group can benefit the most from genetic testing, it is difficult for people with disabilities to fully utilize these services. Therefore, it is necessary to consider ways to subsidize the cost of testing so that people with disabilities can easily undergo genetic testing according to their medical needs. Furthermore, in Korea, there is a stigma surrounding disability. Some parents try to postpone genetic testing because they believe that disability can affect their child’s prospects of marriage. In order to address this, the predominantly negative perception of disabilities in Korean society must be transformed. Thus, it is necessary to address these issues in the “Education for Disability Awareness” program propagated by the government [22,23,24].

Although this study was conducted among specialists with experience in providing genetic testing services to people with disabilities, our findings cannot be generalized, as the sample size was small. However, it is of significance because the study examined the difficulties of people with disabilities regarding genetic testing from the physician’s perspective; this is the first study to do so in Korea. Moreover, it outlined the factors required for cooperative decision-making between the patient and physician. Further research should be conducted among people with disabilities and their families to identify measures to increase accessibility to genetic testing among this population.

## 5. Conclusions

An auxiliary tool that explains the details of genetic testing must be developed to achieve patient-centered medical care through cooperative decision-making between patients with disabilities and physicians. Moreover, a policy should be introduced to reduce medical costs and increase the accessibility of genetic testing for patients with disabilities. It is also necessary to decrease testing costs and thereby reduce the financial burden of patients with disabilities who wish to undergo genetic testing according to their medical needs. Finally, the “Education for Disability Awareness” program in Korea should address the stigma surrounding persons with disabilities, especially among those who are getting married. 

## Figures and Tables

**Table 1:** General characteristics (N = 27).

Major	Specialization	Number of Respondents (Participants, %)	Mean Clinical Experience after Becoming a Specialist (Years)
Department of Rehabilitation	Pediatric Rehabilitation	10 (37.0)	11.3
	Neurorehabilitation	6 (22.2)	13.7
Department of Pediatrics	Pediatric Neurology	8 (29.6)	9.8
	Genomic/Metabolic Disease	1 (3.7)	12
Department of Obstetrics and Gynecology	Maternal/Fetal Care	2 (7.4)	13.5
Total and mean		27 (100%)	11.7

**Table 2:** Survey results.

Questions	N	Yes	No
1. Have you provided medical treatment for a patient with disability in the past 12 months?	27	25 (92.6)	2 (7.4)
2. Have you recommended genetic testing to a patient with disability in the past 12 months?	25 *	24 (96.0)	1 (4.0)
3. Do you use communication tools (video media, easy-to-read brochures, etc.) during genetic testing consultations to help patients make informed decisions?	24 *	12 (50.0)	12 (50.0)
4. Have you faced obstacles while conducting genetic testing for patients with disability and their families?	24 *	24 (100.0)	0 (0.0)

* Question 2 was only answered if the response to question 1 was “yes” and questions 3 and 4 were only answered if the response to question 2 was “yes”. The “no” responses were excluded.

**Table 3:** Usefulness of genetic testing.

Question	N	M ± SD
1. Is genetic testing helpful in providing medical services to patients with disability?	27	4.37 ± 0.69
2. How helpful do you think a genetic diagnosis is for patients with disability?	27	4.11 ± 0.58
3. How helpful do you think a genetic diagnosis is for the families of patients with disability?	27	4.00 ± 0.56
4. Did the information obtained from genetic testing cause a change in the patient’s healthcare?	24 *	3.67 ± 0.82

* The “no” responses were excluded.

## Data Availability

Not applicable.
